# Bio-efficacy of selected long-lasting insecticidal nets against pyrethroid resistant *Anopheles arabiensis* from South-Western Ethiopia

**DOI:** 10.1186/1756-3305-5-159

**Published:** 2012-08-07

**Authors:** Delenasaw Yewhalaw, Abebe Asale, Kora Tushune, Yehenew Getachew, Luc Duchateau, Niko Speybroeck

**Affiliations:** 1 Department of Biology, College of Natural Sciences, Jimma University, Jimma, Ethiopia; 2 Department of Health Service Management, College of Public Health and Medical Sciences, Jimma University, Jimma, Ethiopia; 3 Department of Horticulture, College of Agriculture and Veterinary Medicine, Jimma University, Jimma, Ethiopia; 4 Department of Physiology and Biometrics, Faculty of Veterinary Medicine, Ghent University, Ghent, Belgium; 5 Institute for Health and Society, School of Public Health, Université Catholique de Louvain, Brussels, Belgium

**Keywords:** Bio-efficacy, Long-lasting insecticidal nets, Insecticide resistance, *Anopheles arabiensis*, Ethiopia

## Abstract

**Background:**

The emergence and spread of insecticide resistance in the major African malaria vectors *Anopheles gambiae s.s.* and *Anopheles arabiensis* may compromise control initiatives based on insecticide-treated nets (ITNs) or indoor residual spraying (IRS), and thus threaten the global malaria elimination strategy.

**Methods:**

We investigated pyrethroid resistance in four populations of *An. arabiensis* from south-western Ethiopia and then assessed the bio-efficacy of six World Health Organization recommended long lasting insecticidal nets (LLINs) using these populations.

**Results:**

For all four populations of *An. arabiensis*, bottle bioassays indicated low to moderate susceptibility to deltamethrin (mortality at 30 minutes ranged between 43 and 80%) and permethrin (mortality ranged between 16 and 76%). Pre-exposure to the synergist piperonylbutoxide (PBO) significantly increased the susceptibility of all four populations to both deltamethrin (mortality increased between 15.3 and 56.8%) and permethrin (mortality increased between 11.6 and 58.1%), indicating the possible involvement of metabolic resistance in addition to the previously identified *kdr* mutations. There was reduced susceptibility of all four *An. arabiensis* populations to the five standard LLINs tested (maximum mortality 81.1%; minimum mortality 13.9%). Bio-efficacy against the four populations varied by net type, with the largest margin of difference observed with the Jimma population (67.2% difference). Moreover, there were differences in the bio-efficacy of each individual standard LLIN against the four mosquito populations; for example there was a difference of 40% in mortality of Yorkool against two populations. Results from standard LLINs indicated reduced susceptibility to new, unused nets that was likely due to observed pyrethroid resistance. The roof of the combination LLIN performed optimally (100% mortality) against all the four populations of *An. arabiensis*, indicating that observed reductions in susceptibility could be ameliorated with the combination of PBO with deltamethrin, as used in PermaNet® 3.0.

**Conclusion:**

Our results suggest that bio-efficacy evaluations using local mosquito populations should be conducted where possible to make evidence-based decisions on the most suitable control products, and that those combining multiple chemicals such as PBO and deltamethrin should be considered for maintaining a high level of efficacy in vector control programmes.

## Background

Insecticide-treated nets (ITNs) lead to a reduction of human-vector contact by providing a physical barrier and through insecticidal and/or repellent effects. Wide-scale deployment of ITNs protects users as well as non-users through personal and community level protection gained with high coverage rates [1,2]. In this way, ITNs have been shown to reduce the burden of malaria in pregnant women and young children [3] and reduce the incidence of uncomplicated malarial episodes by around 40% in areas of both stable and unstable malaria relative to untreated nets [4]. Long-lasting insecticidal nets (LLINs) pre-treated with insecticides designed to last the life span of the mosquito net were developed to avoid the need for retreatment every 6 months [5]. To be classified as an LLIN, nets must retain their effective biological activity without re-treatment for at least 20 WHO standard washes under laboratory conditions and three years of recommended use under field conditions [6]. Two techniques have been developed to maintain biological activity: incorporating the insecticide into the textile polymer through extrusion (as with polyethylene and polypropylene), and mixing the insecticide with a wash-resistant resin that is bound around the fibers of the polymer (polyester). Pyrethroids are the only class of insecticide currently recommended to treat mosquito nets. Twelve net types are currently recommended by the WHO Pesticide Evaluation Scheme (WHOPES), and use permethrin, deltamethrin or alpha-cypermethrin, with one combination net using deltamethrin combined with the synergist piperonylbutoxide (PBO) in the roof of the product. However, there are increasing reports of malaria vectors that have developed resistance to the pyrethroids commonly used in LLINs and pyrethroid resistance is now firmly established throughout Africa [7-9]. This resistance to pyrethroids may compromise malaria control as LLINs may lose efficacy, although at present there are no studies linking insecticide resistance to LLIN control failure.

In Ethiopia, ITN use started in 1997 and scaling up commenced in 2005 with the aim of obtaining a high coverage towards effective malaria control. The National Malaria Control Programme (NMCM) distributed 36 million LLINs between 2005 and 2010, targeting 52 million people at risk [10]. Indoor residual spraying has also been conducted using deltamethrin, malathion and bendiocarb.

*An. arabiensis* Patton is the primary malaria vector species in the south-west of the country, and is the only vector species of the *An. gambiae* complex present in the study area. Previous studies within the area indicated that populations of *An. arabiensis* were resistant to DDT, permethrin, deltamethrin, malathion [11,12] and lambdacyhalothrin (D. Yewhalaw *et al*., unpublished). The West African *kdr* mutation (L1014F) was the underlying resistance mechanism observed in these mosquito populations with an allelic frequency of over 98% [11,12]. However, the relationship between *kdr* frequency and phenotypic resistance remains poorly defined; for instance, rapid increases in *kdr* frequency in *An. gambiae s.s.* from western Kenya were not linked to concurrent increases in phenotypic resistance [13]. Moreover, despite *kdr* reaching fixation, LLINs appeared to remain effective. Thus, observed resistance in *An. arabiensis* in the study area may not be solely attributable to target-site resistance, though investigations of other mechanisms have been lacking due to limited capacity to conduct biochemical assays on fresh field-collected specimens, which is required for detection of upregulated esterases, oxidases or GSTs. Furthermore, little is known about the implications of any observed resistance on the anticipated bio-efficacy of insecticidal interventions such as LLINs.

Therefore, this study was conducted to: 1) monitor insecticide resistance and assess the presence of resistance mechanisms other than *kdr* in these mosquito populations and 2) determine the bio-efficacy of six WHOPES-recommended LLINs against pyrethroid resistant populations of *An. arabiensis* from south-western Ethiopia.

## Methods

### Study area and period

Mosquitoes were collected from villages located in Jimma, TiroAfeta, OmoNada and Kerssa districts (*weredas*) in south-western Ethiopia, from November 2011 to January 2012. TiroAfeta, Omo Nada and Kerssa districts are located approximately 255 to 297 km southwest of the capital Addis Ababa, whereas Jimma is located 335 km southwest of the capital. The study area lies between latitudes 7°42’50”N and 07°53’50”N and between longitudes 037°11’22”E and 037°20’36”E, at an altitude of 1,672–1,864 m above sea level. The area has a sub-humid, warm to hot climate, receives between 1,300 and 1,800 mm of rain annually and has a mean annual temperature of 19°C. The rainfall pattern of the area is similar to other parts of Ethiopia, with the long rainy season starting in June and extending up to September while the short rainy season begins in March and extends to April/May. The main socio-economic activities of the local communities in the 3 districts (TiroAfeta, Omo Nada and Kerssa) are mixed farming involving the cultivation of staple crops (maize, teff and sorghum), and cattle and small stock-raising.

Previous assessments showed that *An. arabiensis* was the predominant species present in the area, and populations from all four sites exhibited high resistance to DDT (0–2.7% mortality) in WHO susceptibility tests [11]. Resistance to pyrethroids was also noted for all populations, with mortalities of 10.0, 4.5, 37.3 and 42.7% after exposure to permethrin and 55.5, 56.9, 53.6 and 78.6% after exposure to deltamethrin for *An. arabiensis* populations from Jimma, Omo Nada, Kerssa and TiroAfeta, respectively. Resistance to malathion (60.0–81.8% mortality) but susceptibility to propoxur (99.1–100% mortality) was also noted. Very high (95–100%) allelic frequencies of *kdr-*L1014F mutation were found in all four populations but the ace-1^R^ mutation was not detected [11].

### Mosquito collections

Adult female mosquitoes were collected from inside houses and cow sheds by two teams of two people from 5:00 h to 7:30 h using a torch and aspirator in each of the study districts. Adults were transported to the Vector Biology Laboratory, Asendabo for direct use in CDC bottle assays.

Mosquito larvae were collected from different breeding habitats in the four districts, transported to the Vector Biology Laboratory, Asendabo and were reared to adult stage feeding on dog biscuits and baker’s yeast for use in WHO cone bioassays. All adult mosquitoes were identified morphologically using standard taxonomic keys [14].

### CDC bottle assays

CDC bottle assays were carried out on populations of *An. arabiensis* from the four study districts in order to monitor susceptibility to permethrin and deltamethrin. The bottle assay was conducted following standard procedures [15,16]. Reagent bottles (Wheaton bottles, 250 ml) were coated with 1 ml of either permethrin (21.5 μg/bottle) or deltamethrin (12.5 μg/bottle), which were diluted with factory-grade acetone. Assays with both insecticides were also run following a pre-exposure step in which mosquitoes were exposed to the synergist piperonylbutoxide (PBO, 400 μg/bottle) for one hour before undergoing the standard bottle assays. Each bottle was rolled and inverted in such a way that all interior surfaces were exposed to the solution as the acetone was allowed to evaporate. The bottles and caps were inverted on paper over night in a dark cabinet. Approximately 10–15 field collected adult mosquitoes were introduced into each bottle by mechanical aspiration at time = 0 and mortality was recorded at 15 minutes intervals up to 120 minutes. Mortality was recorded for mosquitoes that could not rest the right way up or fly when the test bottles were slowly rotated. After 120 minutes, mosquitoes were transferred to recovery cups and observed 24 hour later. Mortality after 30 minutes (the resistance threshold for deltamethrin and permethrin in our test conditions) and 24 hour recovery were recorded. Each test had 4 replicates with approximately equal numbers of mosquitoes that were introduced into control bottles coated with acetone only; assays were run simultaneously. For the pre-exposure step, an equal number of mosquitoes were concurrently exposed in a bottle coated with acetone only.

### LLIN sample preparation and chemical assays

Three rectangular nets of 6 net types plus untreated nets to be used as a negative control were purchased from the local market in Uganda due to availability. The production date and batch number of all nets were recorded. For standard LLINs (Olyset®, Netprotect®, Interceptor®, Yorkool® and PermaNet® 2.0), three sub-samples per net were taken and prepared for cone tests by cutting 30 cm x 30 cm pieces: one from the roof and two others with one from each long side of the net. For the combination net PermaNet® 3.0, five sub-samples were prepared for cone tests: one piece from the roof, two samples from the upper half of each long side, and two samples from the lower half of each long side of the net. This was done to verify if there were any differences in bio-efficacy between the lower border region of the sides of the net and the upper region of the sides of the net. Three or five sub-samples were similarly taken adjacent to cone test sub-samples to be used as reference samples in chemical assays. Each sub-sample was rolled up in new aluminium foil, labelled (by net type, net number and sample area) and kept individually in a refrigerator prior to assays. Reference samples were tested for chemical content at an ISO IEC 17025-accredited laboratory to confirm that all nets were within product target doses. For deltamethrin, normal-phase high performance liquid chromatography (HPLC) was conducted as per standard protocols (CIP 333/LN (M)). For alpha-cypermethrin, extraction was conducted with n-hexane and 1,4-dioxane (95:5 v/v) with the mixture then shaken and sonicated and filtered on a 0.45 mm teflon membrane, whereas for permethrin hot xylene extraction was followed by drying, reconstitution and filtration, with both then assessed via HPLC. The precision as measured by the Relative Standard Deviation was 0.79% and 1.79%, respectively and the recovery was 101 and 102%, respectively.

### WHO cone bioassays

For each individual sub-sample prepared for cone tests from both standard LLINs and the combination LLIN, four cone tests were conducted at a time following standard WHO procedure [6] using mosquitoes from each collection district. Five non-blood fed two to three day old adult female *An. arabiensis* were introduced into each cone and exposed to each bed net sample for 3 minutes before being transferred to paper cups and held with access to 10% sugar solution. Knockdown (KD) was recorded at 1, 3, 5, 10, 15, 30, 45 and 60 minutes and mortality (MT) was recorded 24 hours post-exposure. A total of 180 mosquitoes were tested for each net type (20 mosquitoes x 3 sub-samples x 3 nets) for standard LLINs while 300 mosquitoes were tested for the combination net (20 mosquitoes x 5 sub-samples x 3 nets) for each of the four mosquito populations. Replicates of cone assays with sub-samples taken from untreated nets were also conducted concurrently as a negative control. Mortality was corrected using Abbott’s formula when mortality in the control exceeded 5% [17]. Bioassays were carried out at 27 ± 2°C and 80 ± 4% relative humidity.

### Data analysis

Data were analysed using SAS software package. Association between % knockdown and % mortality by site, type of net and net section were assessed vialine arregression. Differences in mean % mortality for the sections of specific net types were assessed via Student’s *t*-test for standard LLINs and via ANOVA for the combination net. Variations in mean % mortality between the 5 net types, and for each net type between the 4 mosquito populations, were assessed via ANOVA with Duncan’s method applied to identify groupings. The alpha value was set at 0.05 with P < 0.05 considered significant in the analysis.

## Results

### Bottle bioassays

**Figure 1:**
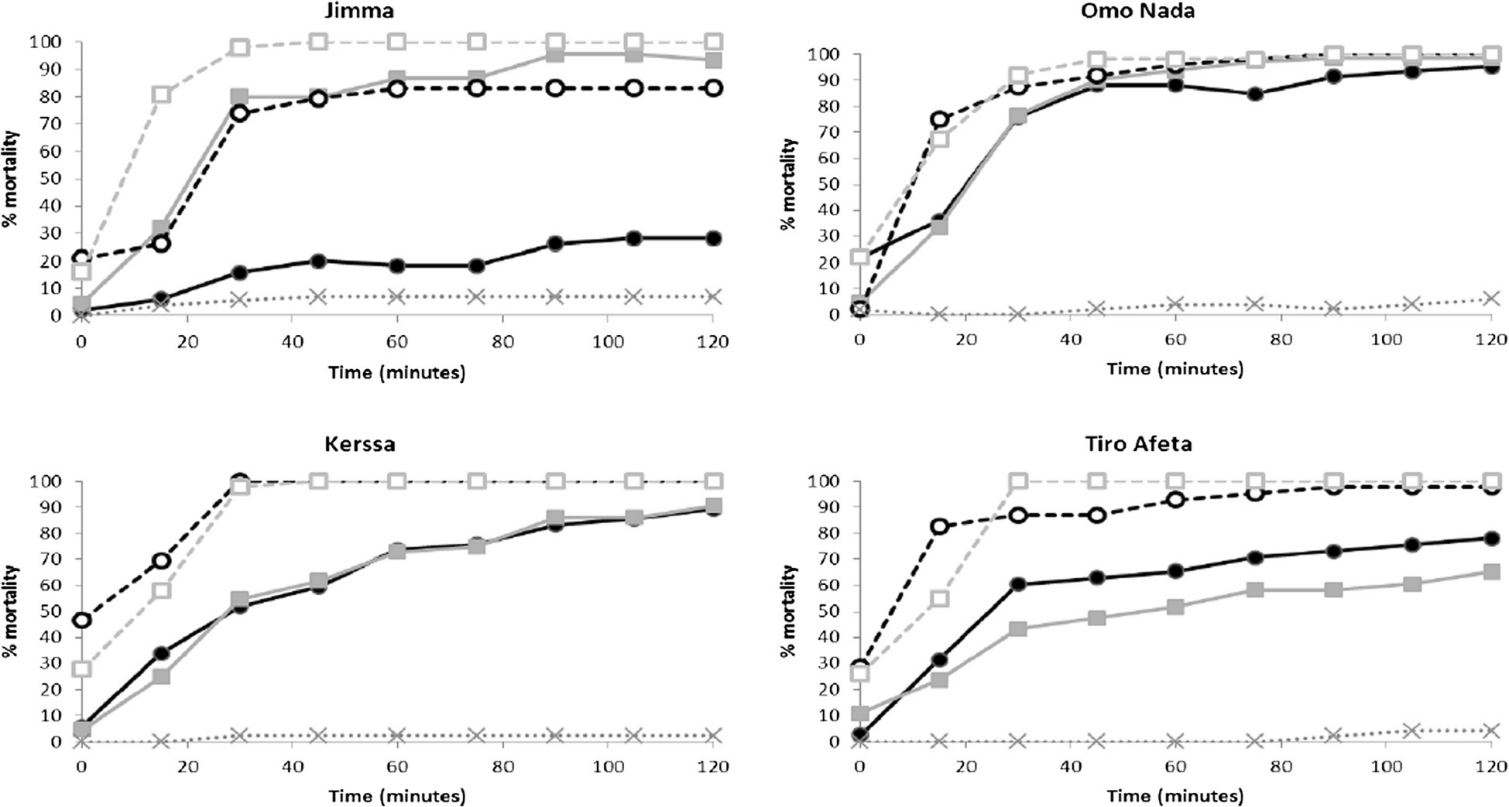
**Susceptibility of populations of *An. arabiensis *adult female mosquitoes collected from four sites in Ethiopia to permethrin alone (black filled circle), permethrin following 60 mins pre-exposure to PBO (black unfilled circle), deltamethrin alone (grey filled square), and deltamethrin following 60 mins pre-exposure to PBO (grey unfilled square) in bottle bioassays.** Average of all controls is also indicated (x).

Results of the susceptibility status of populations of *An. arabiensis* from the 4 collection sites as determined in CDC bottle bioassays are presented in Figure 1. At the 30 minute diagnostic period, all four populations showed low to moderate susceptibility to deltamethrin (mortality ranged between 43% and 80%) and permethrin (mortality ranged between 16% and 76%). Susceptibility to deltamethrin was highest for the Jimma and Omo Nada populations (79.7 and 76.5% mortality, respectively), though susceptibility to permethrin was highest for the Omo Nada population only (75.9% mortality) with mortality %  ≤ 60% for all other situations. The synergist PBO reduced the expression of deltamethrin and permethrin resistance in the four populations of *An. arabiensis*. Following pre-exposure for 1 hour to PBO, the susceptibility of mosquito populations increased at all four sites to both deltamethrin (mortality increased from 18.0 to 56.8%, to range from 91.8 to 100%) and permethrin (mortality increased from 11.6 to 58.1% to range from 73.9 to 100%). The increase in mortality following exposure to PBO was greatest at Jimma and TiroAfeta for deltamethrin and at Kerssa and TiroAfeta for permethrin, however for the Jimma population there was not such a marked increase in susceptibility to permethrin following pre-exposure to PBO with mortality remaining relatively low (73.9%).

### Cone bioassays

**Figure 2:**
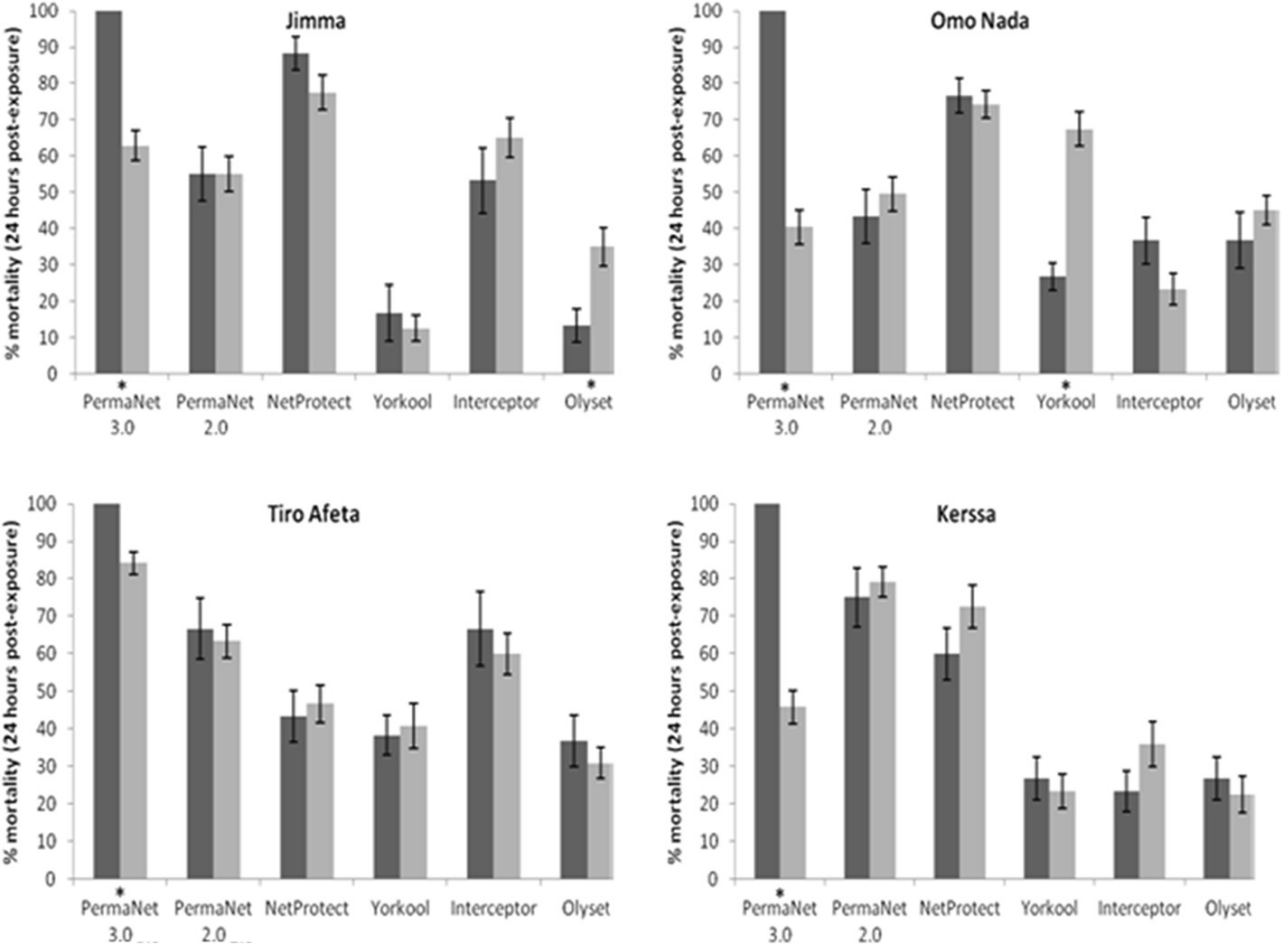
**Bio-efficacy of roof (black rectangles) and side (grey rectangles) samples of six long-lasting insecticidal net types against *An. arabiensis *adult female mosquitoes collected from four sites in Ethiopia following 3-minutes exposure in standard WHO cone bioassays.** Bars show mean percentage mortality ± standard error, asterisks indicate significant difference detected between roof and sides (P < 0.05).

Overall, there was a significant relationship between % knockdown and % mortality (R^2^ = 0.53, n = 959, p < 0.001), noting that one data point (single sample of PermaNet® 2.0 side) was missing from the bio-efficacy data set. When data were stratified by site and net type, there was a significant association between mean % knockdown and % mortality for PermaNet® 3.0, Interceptor® and Olyset® against all mosquito populations (p < 0.05) (Figure 2). For PermaNet® 2.0, Netprotect® and Yorkool®, there was an association between mean % knockdown and % mortality for two populations only, although there was no consistency in populations where an association was found. Based on observed associations, further assessments of bio-efficacy focused on mortality data.

When mean % mortality was compared between the different net sections for each net type for each study site, there were significant differences observed in the bio-efficacy of net sections for PermaNet® 3.0 against all four populations (p < 0.05 for all), for Olyset® against the Jimma population (p = 0.012) and for Yorkool® against the Omo Nada population (p < 0.05). However, for PermaNet® 3.0 there was a clear grouping of lower and upper side data (mortality of 59.2 and 66.7%, respectively), with roof data significantly higher (100%). Based on observed associations, data for sections of the specific net types were grouped together except for PermaNet®3.0 for which the roof and side panels were assessed separately.

Although there was an association between mean % knockdown and % mortality for 16 of the 40 other net types–net section–collection site groupings, there was no observable pattern. There was a particularly large disparity in the mean % knockdown and mortality data for Yorkool® roof sections against the Omo Nada *An. arabiensis* population.

Table 1 shows the bio-efficacy of the six LLINs tested against the four *An. arabiensis* populations. Bio-efficacy against each population varied significantly between net types: Jimma (F = 39.24, n = 240, p < 0.001); Omo Nada (F = 21.24, n = 239, p < 0.001), Kerssa (F = 34.21, n = 240, p < 0.001); TiroAfeta (F = 28.73, n = 240, p < 0.001). The greatest variation in bio-efficacy was observed for the Jimma population (PermaNet® 3.0 roof: 100%, Yorkool®: 13,9%), with the least variation observed against the TiroAfeta population (PermaNet® 3.0 roof: 100%, Yorkool®: 40.0%).

**Table 1:** **Bio-efficacy (in mean percentage mortality) of samples of six long-lasting insecticidal net types against *An. arabiensis *adult female mosquitoes collected from four sites in Ethiopia following 3-minutes exposure in standard WHO cone bioassays**

	**Net type/section**							
**Collection site**	**PermaNet**® **3.0**		**PermaNet**® **2.0**	**NetProtect**®	**Yorkool**®	**Interceptor**®	**Olyset**®	**F statistic;**
	**Roof**	**Side**						**P-value**
**Jimma**	100a	62.92c	55.00c	81.11b	13.89e	61.11c	27.78d	39.24;
								<0.0001*
**Omo Nada**	100a	40.42c,d	47.43c	75.00b	53.89c	27.78d	42.22c	21.24;
								<0.0001*
**Kerssa**	100a	45.83c	77.78b	68.33b	24.44d	31.67d	23.89d	34.21;
								<0.0001*
**TiroAfeta**	100a	84.17b	64.44c	45.56d	40.00d	62.22c	32.78d	28.73;
								<0.0001*

*Differences in mean % MT between net types at a specific collection site were significant (p < 0.05; ANOVA and Duncan’s test); Means within a row followed by the same letter (s) are not significantly different from each other (p ≥ 0.05).

The bio-efficacy of the roof section of PermaNet® 3.0 was consistently high against all mosquito populations (all 100%). Apart from this, the bio-efficacy of each specific net type varied significantly between mosquito populations: PermaNet® 3.0 sides (F = 22.78, n = 192, p < 0.001); PermaNet® 2.0 (F = 11.11, n = 143, p < 0.001); Netprotect® (F = 16.83, n = 144, p < 0.001); Yorkool® (F = 18.70, n = 144, p < 0.001); Interceptor® (F = 17.37, n = 144, p < 0.001); Olyset® (F = 4.34, n = 144, p < 0.0058). This indicates that with the exception of the combination roof of PermaNet® 3.0, the standard LLINs performed differently against the different *An. arabiensis* populations.

Target insecticide and/or synergist concentrations for all LLINs fell within manufacturer specifications (Table 2).

**Table 2:** Mean (± standard error) insecticidal or synergist concentration and % as proportion of target concentration for roof and side samples from six different LLINs types as determined via high performance liquid chromatography

		**Target dose**		**Roof**	**Side**
**Net type**	**Chemical**	**Mean**	**Range**	**Mean**	**Mean**
PermaNet® 3.0	Deltamethrin	2.8 g/kg (sides)	2.1–3.5	n/a	2.4 ± 0.1
		4 g/kg (roof)	3.0–5.0	3.8 ± 0.1	n/a
	Piperonylbutoxide	25 g/kg (roof)	18.75–31.25	24.3 ± 1.0	n/a
PermaNet®2.0	Deltamethrin	55 mg/m2	41.25–68.75	60.8 ± 1.0	62.5 ± 4.1
NetProtect®	Deltamethrin	1.8 g/kg	1.35–2.25	1.9 ± 0.0	1.9 ± 0.0
Yorkool®	Deltamethrin	55 mg/m2	41.25–68.75	56.2 ± 8.3	59.9 ± 9.4
Inteceptor®	Alpha-cypermethrin	200 mg/m2	150.0–250.0	223.6 ± 20.8	196.0 ± 33.7
Olyset®	Permethrin	20 g/kg	17.0–23.0	22.4 ± 0.1	22.2 ± 0.1

## Discussion

Bottle bioassays revealed that populations of *An. arabiensis* from all four localities in south-western Ethiopia had low to moderate susceptibility to both permethrin and deltamethrin for the diagnostic dose and time used. Although no historical data for the same populations or reference data from a susceptible *An. arabiensis* strain were available, previous WHO susceptibility tests also indicated reduced susceptibility of mosquito populations from the same study area to these insecticides [11,12]. Moreover, the susceptibility of mosquito populations to both permethrin and deltamethrin increased significantly when synergized by PBO, suggesting the presence of metabolic-based resistance mechanisms. Since PBO inhibits two major metabolic systems (P450s and non-specific esterases) that are otherwise responsible for degrading or sequestering the insecticide [18] and also enhances cuticular penetration thereby increasing the rate of uptake into the mosquito [19], it is difficult to know which mechanisms are operating without conducting a battery of other tests such as esterase-only synergist biochemical assays or genetic analyses. This was beyond the scope of this initial evaluation but further investigations of resistance mechanisms are clearly warranted to better define and quantify resistance mechanisms present in the test populations and verify the preliminary evidence of metabolic-based mechanisms as indicated by bottle bioassays.

Low knockdown and mortality of the four *An. arabiensis* populations following exposure to standard LLINs may be explained by either limited bioavailability of active ingredient on the LLIN surface or by physiological resistance of mosquitoes to the insecticide. Chemical assays indicated that pyrethroid content was satisfactory for all LLIN types, and as nets were new and had not been washed it was assumed that surface chemical content was satisfactory. It was most likely that reductions in efficacy were due to previously-identified *kdr* mutations and/or suspected metabolic resistance mechanisms. This was supported by the observed bio-efficacy of the roof of PermaNet 3.0, since the deltamethrin and PBO combination clearly restored optimal bio-efficacy against all four populations. While loss in efficacy of pyrethroid ITNs has been associated with high *kdr* mutation frequency in *An. gambiaes.s.* in Burkina Faso [20], in Western Kenya a high *kdr* frequency was not associated with a reduction in ITN efficacy [13]. General consensus among experts is that metabolic resistance is considered more of a threat than *kdr*, with major loss of efficacy of permethrin-treated nets in experimental huts associated with oxidase-based metabolic resistance in *An. gambiae* in Cameroon [21] and *An. arabiensis* in Cameroon [20]. Co-occurrence of *kdr* and P450- based resistance has been reported in mosquito populations from several countries [22,23], leading to extremely high levels of pyrethroid resistance [24,25] and extreme reduction in LLIN efficacy against *An. gambiae* in Akron, Benin [20]. The likely co-existence of multiple resistance mechanisms in *An. arabiensis* from the four areas in Ethiopia and the observed significant reductions in their susceptibility to LLINs in cone bioassays raises major concerns for the performance of pyrethroid interventions in Ethiopia.

In Ethiopia, DDT has been extensively used in indoor residual spraying (IRS) in alternation with malathion for over five decades. ITN use started in 1997 with significant scale up since 2005 (mainly LLINs) with the aim of obtaining a high coverage towards upgraded malaria control. In addition, pyrethroids (deltamethrin) were used in indoor residual spraying in 2009 [26]. The prolonged use of DDT and malathion, the high coverage of LLINs and the recent use of pyrethroids for indoor residual spraying are likely to have enhanced the selection pressure for insecticide resistance in the *An. arabiensis* populations in Ethiopia. The increasing trend in use of pyrethroid for indoor residual spraying may not be consistent with the need to preserve the effectiveness of LLINs [26]. Trape *et al*. [27] also reported that LLINs may result in mosquito resistance to insecticides and that the increase in pyrethroid resistance of *An. gambiae* likely caused the rebound of malaria morbidity in Senegal. In 2011, Ethiopia switched from pyrethroids (deltamethrin) to carbamates (bendiocarb) for IRS because of resistance reported to other classes of insecticides [28]. The carbamate class is the only class of insecticides to which these mosquito populations are susceptible in Ethiopia. Unfortunately, evidence of resistance to carbamates (bendiocarb) has also emerged in Afro-tropical malaria vectors from elsewhere [29-33].

If resistance and control failure is shown to both pyrethroids and DDT, programs will need to consider carbamates and organophosphates [34]. High levels of control have been achieved with certain carbamates and this insecticide class has been evaluated for potential use on ITNs [35]. However, safety remains a concern with carbamates, and formulations with low toxicity or methods of delivery that limit human contact may be potential options alone or in combination with pyrethroid-treated nets [36]. Combining two classes of insecticides on nets may also present a method for managing resistance, by exposing mosquitoes to two insecticides with different modes of action [37,38]. However, since there are currently no non-pyrethroid LLINs available combining these insecticides with a synergist such as PBO offers a viable and readily-available alternative to standard LLINs for areas with pyrethroid-resistant *Anopheles* populations.

While cone bioassays on new nets are by no means a definitive indication of anticipated net performance under field conditions, these assays can provide valuable comparative information across numerous sites, where experimental huts are not available. Non-uniformity of nets such as PermaNet® 3.0 complicate evaluations where net sections are assessed separately; since anophelines most frequently make contact with the roof of bed nets (37, P. McCall personal communication), emphasis would be well placed on outcomes from roof sections*.* Further studies are warranted to investigate the impact of observed resistance on LLIN bio-efficacy, and also to better define the relationship between results from cone bioassays, experimental hut trials and real-life use. In Mali, *An. gambiaes.l.* populations from two sites showed no apparent differences in susceptibility to alpha-cypermethrin nets when tested in laboratory cone bioassays yet one population showed reduced susceptibility to the same nets in experimental hut trials [39].

This study was the first attempt to establish the comparative bio-efficacy data of six types of WHO-recommended LLINs against pyrethroid resistant populations of *An. arabiensis* from Ethiopia. Although comparisons to a susceptible strain were not incorporated due to logistical limitations, the low bio-efficacy of new LLINs against these populations suggests that the standard LLINs tested would have sub-optimal efficacy under field conditions. We also report for the first time the likely existence of metabolic resistance in addition to *kdr* mutations in Ethiopia. The underlying mechanisms involved in metabolic resistance should be further assessed using esterase and glutathione-S-transferase synergists as well as at the genetic level using the microarray technique. LLINs should be assessed at additional sites across the country to compare bio-efficacy against populations with different resistance levels or mechanisms, and attempts need to be made to relate results to observed phenotypic resistance and observed or reported LLIN failure.

## Conclusion

Relatively low knockdown and mortality rates were observed for four pyrethroid resistant populations of *An. arabiensis* from south-western Ethiopia following exposure to new, unused WHO-recommended standard LLINs. Conversely, optimal bio-efficacy was observed for the deltamethrin + PBO roof of PermaNet® 3.0 against all four populations. Although the approach used cone bioassays with new nets only, it provided compelling information suggesting that pyrethroid resistance may be a cause for concern for sustained efficacy of pyrethroid-based interventions in Ethiopia. It also indicates the utility of conducting comparative bio-efficacy studies using local mosquito populations, and underscores the urgent need to establish an insecticide resistance management (IRM) strategy for Ethiopia.

## Competing interests

Authors declare that they have no competing interests.

## Authors’ contribution

DY conceived and designed the study, was involved in field supervision and drafted the manuscript; AA & DY were involved in WHO cone bioassays and CDC bottle assays; KT, YG, LD & NS reviewed the manuscript. All authors read and approved the final version of the manuscript.

## References

[B1] Rafinejad J Vatandoosta H Nikpoor F Abai MR Shaeghi M Duchen S Rafi F Effect of washing on the bio-efficacy of insecticide-treated nets (ITNs) and long-lasting insecticidal nets (LLINs) against main malaria vector Anopheles stephensi by three bioassay methods J Vector Borne Dis 2008 45 143 150 18592843

[B2] Gu W Novak RJ Predicting the impact of insecticide –treated bed nets on malaria transmission: the devil is the detail Malaria J 2009 8 256 10.1186/1475-2875-8-256 PMC2780451 19917119

[B3] World Health Organization: Malaria control today Current WHO recommendations 2005 Geneva, Switzerland: Working document Roll Back Malaria Department 75

[B4] Lengeler C Insecticide-treated bed nets and curtains for preventing malaria Cochrane Database of Systematic Reviews 2004, Issue 2 2009 CD000363 10./002/14651858 10.1002/14651858.CD000363.pub2 15106149

[B5] Gunasekaran K Vaidyanathan K Wash resistance of PermaNets in comparison to hand-treated nets Acta Trop 2008 108 154 157 18053964 10.1016/j.actatropica.2007.10.008

[B6] World Health Organization Guideline for laboratory and field testing of long-lasting insecticidal mosquito nets 2005 WHO/CDS/WHOPES/GCDPP/: World Health Organization 11

[B7] Ranson H N’Guessan R Lines J Moiroux N Nkuni Z Corbel V Pyrethroid resistance in African anopheline mosquitoes: what are the implications for malaria control? Trends Parasitol 2011 27 91 98 10.1016/j.pt.2010.08.004 20843745

[B8] Coleman M Sharp B Seocharan I Hemingway J Developing an evidence-based decision support system for rational insecticide choice in the control of African malaria vectors J Med Entomol 2006 43 4 663 668 10.1603/0022-2585(2006)43[663:DAEDSS]2.0.CO;2 16892622

[B9] Hemingway J Ranson H Insecticide resistance in insect vectors of human disease Annu Rev Entomol 2000 45 371 391 10.1146/annurev.ento.45.1.371 10761582

[B10] World Health Organization World Malaria Report 2010 accessed on 23 March 2012 http://whqlibdoc.who.int/publications/2010/9789241564106 eng.pdf

[B11] Yewhalaw D Wassie F Steurbaut W Spanoghe P Van Bortel W Denis L Tessema DA Getachew Y Coosemans M Duchateau L Speybroeck N Multiple insecticide resistance: an impediment to insecticide-based malaria vector control program PLoS ONE 2011 6 1 10.1371/journal.pone.0016066 PMC3020220 21264325

[B12] Yewhalaw D Van Bortel W Denis L Coosemans M Duchateau L Speybroeck N First evidence of high knockdown resistance frequency in *Anopheles arabiensis* (Diptera: Culicidae) from Ethiopia Am J Trop Med Hyg 2010 83 1 122 125 10.4269/ajtmh.2010.09-0738 20595490 PMC2912588

[B13] Mathias DK Ochomo E Atieli F Ombok M Bayoh MN Olang G Muhia D Kamau L Vulule JM Hamel MJ Hawley WA Walker ED Spatial and temporal variation in the kdrallele L1014S in *Anopheles gambiaes.s.* and phenotypic variability in susceptibility to insecticides in Western Kenya Malaria J 2011 10 10 10.1186/1475-2875-10-10 PMC3029224 21235783

[B14] Gillies MT Coetzee M A Supplement to the Anophelinae Africa south of the Sahara (Afrotropical Region) 1987 Johannesburg, South Africa: South African Institute for Medical Research 55

[B15] Brogdon WC McAllister JC Insecticide resistance and vector control Emerg Infect Dis 1998 4 605 613 10.3201/eid0404.980410 9866736 PMC2640263

[B16] Center for Disease Control and Prevention: Evaluating mosquitoes for insecticide resistance. Atlanta, GA Center for Disease Control and Prevention 2012 http://www.cdc.gov/ncidod/wbt/resistance/assay/bottle/index.htm

[B17] Abbott WS A method for computing the effectiveness of insecticides J Econ Entomol 1925 18 265 267

[B18] Ahmed M Denholm I Bromilow RH Delayed cuticular penetration and enhanced metabolism of deltamethrin in pyrethroid resistant strains of *Helicoverpaarmigera* from China and Pakistan Pest Management Science 2006 62 9 805 810 10.1002/ps.1225 16649192

[B19] Moores G Bingham G Use of temporal synergism to overcome insecticide resistance Outlooks on Pest Management 2005 16 1 7 9 10.1564/16feb03

[B20] Corbel V Chabi J Dabire RD Etang J Nwane P Pigeon O Akogbeto M Hougard JM Field efficacy of a new mosaic long-lasting mosquito net (PermaNet 3.0) against pyrethroid-resistant malaria vectors: a multi-centre study in Western and Centeral Africa Malaria J 2010 9 113 10.1186/1475-2875-9-113 PMC2877060 20423479

[B21] Etang J Chandre F Guillet P Manga L Reduced bio-efficacy of permethrin EC impregnated bed nets against *Anopheles gambiae* strain with oxidase-based pyrethroid tolerance Malaria J 2004 3 46 10.1186/1475-2875-3-46 PMC538265 15569394

[B22] Djouaka RF Bakare AA Coulibaly ON Akogbeto MC Ranson H Hemingway J Expression of the cytochrome P450s, CYP6P3 and CYP6M2 are significantly elevated in multiple pyrethroid resistant populations of *Anopheles gambiaes.s.* from southern Benin and Nigeria BMC Genomics 2008 9 538 10.1186/1471-2164-9-538 19014539 PMC2588609

[B23] Muller P Warr E Stevenson BJ Pignatelli PM Morgan JC Steven A Yawson AE Mitchell SN Ranson H Hemingway J Field-caught permethrin-resistant *Anopheles gambiae* overexpress CYP6P3, a P450 that metabolizes pyrethroids PLoS Genet 2008 4 e1000286 10.1371/journal.pgen.1000286 19043575 PMC2583951

[B24] Hardstone MC Leichter CA Scott JG Multiplicative interaction between the two major mechanisms of permethrin resistance, *kdr* and cytochrome P450-monooxygenase detoxification in mosquitoes J EvolBiol 2009 22 416 423 10.1111/j.1420-9101.2008.01661.x 19196389

[B25] Berticat C Bonnet J Duchon S Agnew P Weill M Corbel V Costs and benefits of multiple resistance to insecticides for *Culexquinquefasciatus* mosquitoes BMC EvolBiol 2008 8 104 10.1186/1471-2148-8-104 PMC2359736 18397515

[B26] World Health Organization The technical basis for co-ordinated action against insecticide resistance: preserving the effectiveness of modern malaria vector control 2011 Geneva, Switzerland: World Health Organization

[B27] Trape GF Tall A Diagne N Ndiath O Ly AB Faye J Dieye-Ba F Roucher C Bouganali C Badiane A Sarr FD Mazenot C Toure-Balde A Druile P Puijalon OM Rogier C Sokhna C Malaria morbidity and pyrethroid resistance after the introduction of insecticide-treated bed nets and artemesinin-based therapies: a longitudinal study Lancet Infect Dis 2011 11 2 925 932 10.1016/S1473-3099(11)70194-3 21856232

[B28] Van den Berg H Zaim M Yadav RS Soares A Ameneshewa B Mnzava A Hii J Dash AP Ejov M Global trends in the use of insecticides for vector-borne disease control Environmental Health Perspectives 2012 http://dx.doi.org/10.1289/ehp.11o4340 10.1289/ehp.1104340 PMC3339467 22251458

[B29] Hunt RH Fuseini G Knowles S Stiles-Ocran J Verster R Kaiser ML Insecticide resistance in malaria vector mosquitoes in four localities in Ghana, West Africa ParasitVectors 2011 16 107 10.1186/1756-3305-4-107 PMC3145582 21679391

[B30] World Health Organization World Malaria Report 2011 Geneva, Switzerland: World Health Organization http://www.who.int/malaria/world malaria report 2011/WMR

[B31] Hunt RH Edwardes M Coetzee M Pyrethroid resistance in South African *Anopheles funestus* extends to Likoma Island in Lake Malawi ParasitVectors 2010 3 12 10.1186/1756-3305-3-122 PMC3020165 21192834

[B32] Ranson H Abdallah H Badolo A Guelbeogo WM Kerah-Hinzoumbe C Yangalbe-Kalnone E Sagnon NF Simard F Coetzee M Insecticide resistance in *Anopheles gambiae*: data from the first year of multi-country study highlight the extent of the problem Malaria J 2009 8 299 10.1186/1475-2875-8-299 PMC2804687 20015411

[B33] Vezenegho SB Brooke BD Hunt RH Coetzee M Koekemoer LL Malaria vector composition and insecticide susceptibility status in Guinea Conakry, West Africa Med Vet Entomol 2009 23 326 334 10.1111/j.1365-2915.2009.00840.x 19941598

[B34] President’s Malaria Initiative Guidelines for entomological monitoring and insecticide resistance management 2011 President’s Malaria Initiative 7

[B35] Kolaczinski JH Fanello C Herve JP Conway DJ Carnevale P Curtis F Experimental and molecular genetic analysis of the impact of pyrethroid and non-pyrethroid insecticide impregnated bed nets for mosquito control in an area of pyrethroid resistance Bulletin Entomol Res 2000 90 125 132 10.1017/s0007485300000237 10948372

[B36] Kulkarni M Malima R Mosha FW Msangi S Mrema E Kabula B Lawrence B Kinung’hi S Swilla J Kisinza W Rau ME Miller JE Schellenberg JA Maxwell C Rowland M Magesa S Drakeley C Efficacy of pyrethroid-treated nets against malaria vectors and nuisance-biting mosquitoes in Tanzania in areas with long-term insecticide-treated net use Trop Med Inter Health 2007 12 9 1061 1073 10.1111/j.1365-3156.2007.01883.x 17875017

[B37] Guillet P N’Guessan R Darriet F Traore-Lamizana M Chandre F Carnevale P Combined pyrethroid and carbamate ‘two-in-one’ treated mosquito nets: field efficacy against pyrethroid resistant *Anopheles gambiae* and *Culexquinquefasciatus* Med Vet Entomol 2001 5 105 112 11297094 10.1046/j.1365-2915.2001.00288.x

[B38] Hougard JM Corbel V N’Guessan R Darriet F Chandre F Akogbeto M Baldet T Guillet P Carnevale P Traore-Lamizana M Efficacy of mosquito nets treated with insecticide mixtures or mosaics against insecticide resistant *Anopheles gambiae* and *Culexquinquefasciatus* (Diptera: Culicidae) in Cote d’Ivoire Bulletin Entomol Res 2003 93 491 498 10.1079/ber2003261 14704095

[B39] Fane M Cisse O Sekou C Traore F Sabatier P *Anopheles gambiae* resistance to pyrethroid-treated nets in cotton versus rice areas in Mali Acta Trop 2011 22 1 1 6 22154879 10.1016/j.actatropica.2011.11.013

